# Notes on Health Resorts

**Published:** 1899-12-02

**Authors:** Robert Saundby


### NOTES ON HEALTH RESORTS.

### MARTIGNY, CONTREXÉVILLE, AND VITTEL.

By Robert Saundby, M.D., LL.D., F.R.C.P.

The department of the Vosges is little known to English tourists, and indeed does not offer much to attract them. It forms part of the ancient Duchy of Lorraine, that part, in fact, which lies nearest to the annexed portion of the province. Its so-called mountains are mostly broad topped hills upon which oak forests alternate with wide stretches of cultivated land, and on the whole the country is rather prosperous than picturesque. Pure and fresh air abounds, but it is not a land to draw tourists to it by any special beauty of scenery. The strategical railway from Langres to Nancy runs through these broad hills, and passes the three stations whose names are written at the head of this paper in the order in which they are seen by the traveller coming from Langres. They are close together, only eight miles separating Martigny from Contrexéville, while Vittel is three miles further on. All these villages—for they are nothing more—have acquired distinction from the possession of mineral waters, and the chief of them—Contrexéville—has become so well known that every season thousands of patients come from all parts of the world to seek health in its chalky valley. The railway service from Paris is direct, there being through carriages with restaurant cars attached, and the journey is accomplished in about seven and a-half hours. It is also possible, but not advisable, to go direct from London *viâ* Calais, Chalons, and Nancy, leaving London by the night mail, and arriving between three and four o'clock in the afternoon of the following day.

### Martigny-les-Bains.

In the direct journey from Paris Martigny is the first reached of these three stations, which have been rendered more or less famous by the presence of the peculiar water of the district; it lies nearly 1,200 feet above the sea level. The valley is bounded by gently sloping hills, so that the two hotels and casino are pleasantly situated upon rising ground. They have a good south-westerly exposure, and the air is fresh and bracing; in fact, the dry soil and favourable aspect of Martigny, apart from the virtues of its mineral waters, would entitle it to be considered a health resort. The therapeutic properties of the waters have been known locally since 1767, but it was not until 1859 or 1860 that any provision was made for visitors from a distance, and the first hotel and Etablissement were built. Within the last two years the place has passed under the control of a company, which has erected a new and sumptuous hotel, and has greatly beautified and improved the park and Etablissement. At the present time visitors may be assured that they will find Martigny a thoroughly well-appointed watering place. The hotels are not only good, but reasonable in their charges. The waters are derived from the springs, viz.: Sources Nos. 1 and 2, and La Savonneuse, the last being used as its name suggests, for external purposes only.

Analyses of the Martigny Waters.

| | | Source No. 1. Per mille. | Source No. 2. Per mille. | La Savonneuse. Per mille. |
|---|---|---|---|---|
| Free carbonic acid gas | | Traces. | Traces. | — |
| Bicarbonate | of soda | 0,0160 | 0,0120 | 0,1520 |
| | of magnesia | 0,1750 | 0,1800 | 0,1300 |
| | of lime | 0,1620 | 0,1580 | 0,3870 |
| | of lithia | 0,0320 | 0,0190 | Traces |
| | of iron | 0,0040 | 0,0310 | Traces |
| Sulphates | of soda | 0,2290 | 0,2360 | 0,0210 |
| | of magnesia | 0,3300 | 0,3340 | 0,1500 |
| | of lime | 1,4240 | 1,4400 | 0,5200 |
| Chlorides | of sodium | 0,0950 | 0,1050 | 0,0600 |
| | of potassium | 0,0120 | 0,0140 | — |
| Phosphate of lime | | 0,0028 | 0,0019 | — |
| Silicate of soda | | 0,0532 | 0,0456 | — |
| Silicate of lime | | 0,0029 | 0,0014 | — |
| Fluorine, arsenic, borax, manganese, alumina, and organic matter | | 0,1141 | 0,0681 | Traces (of arsenic, silica, and alumina) |

Source No. 1 is that used for urinary and biliary lithiasis, glycosuria, gouty affections of the bladder, pyelitis, gout, rheumatism, &c. Source No. 2 is a blood tonic, and is also employed in uterine disorders. La Savonneuse is for external use only. According to Professor Jacquemin, of Nancy, the substance that gives the peculiar soapiness to the water is silicate of alumina. The spring is cold, and needs to be heated for use to a temperature of 34 C. (93·2 F.). Excellent arrangements exist for baths, douches, douche-massage, and massage under water, as practised at Aix and Vichy.

Dr. Liegois has pointed out that Martigny is extremely suitable for the treatment of diseases of the heart and arteries. It has a dry, bracing climate, and a sheltered situation, while its mineral waters seem well adapted for this purpose. The suggestion is a very good one. There should be no difficulty in providing all that is useful in the Nauheim course, and if the right man can be found to work out the idea, Martigny has a future of prosperity before it. In the present struggle with its neighbours the advantages do not lie upon its side, and there would be much wisdom in the adoption of a line of its own. The season is from the middle of May until the third week in September, and the resident physicians are Drs. Dedet, Huguat, and Lafosse.

### Contrexéville.

Eight miles beyond Martigny the train stops in a narrow valley, at the bottom of which runs the little river Vair, along whose banks is the village of Contrexéville. The Etablissement, with pump-room and baths, casino, hotel, and dependent villas, is built within the iron railings of a small park, bounded on one side by the river and at its northern end scaling the hillside toward the railway escarpment. The hills rise abruptly from the village, and can be climbed by zigzag paths or winding roads. At the top the land stretches away towards the west in a rolling plateau and the air is fresh and bracing. At the bottom the conditions are not so favourable, the air is hot and oppressive; some artificial pools in the park are pleasant neither to sight nor to smell, and the night air is said to be often damp; but Contrexéville has won its way in the face of these disadvantages. It is 1,026 ft above the level of the sea, and the soil is the same limestone formation as at Martigny, but the great possession of Contrexéville is its water.

The medicinal properties of the waters of Contrexéville were first brought under scientific notice in the year 1760 by Dr. Bagard, of Nancy, chief physician to Duke Stanislas, of Lorraine, ex-King of Poland, and they soon acquired such a reputation that before the Revolution many of the French nobles had erected houses for themselves upon the hills overlooking the village, and a large building, called the Château des Anglais, is said to have been constructed about that time by some English visitors attracted by the fame of the springs. The Revolution put an end to this commencing prosperity, and it was not until after the establishment of the Second Empire that Contrexéville began to regain its lost position, but once restored to notice its success has been permanent, because it is based upon the genuine therapeutic qualities of its unrivalled waters.

When we speak of Contrexéville water the "Pavillon" spring is meant; but there are three other sources—the "Quai," the "Prince," and the "Souveraine." The last is used where a greater aperient action is desired, as it contains less iron and more magnesia. The "Prince" is popularly believed to be of use as a local application in eye diseases, and the "Quai" is sometimes spoken of as the "Source des demoiselles," and is employed in the treatment of chlorotic anæmia.

(*To be continued.*)

